# Reversal of the Temperature Dependence of Hydrophobic Hydration in Supercooled Water

**DOI:** 10.1021/acs.jpclett.1c02399

**Published:** 2021-08-26

**Authors:** Henry S. Ashbaugh

**Affiliations:** Department of Chemical and Biomolecular Engineering, Tulane University, New Orleans, Louisiana 70118, United States

## Abstract

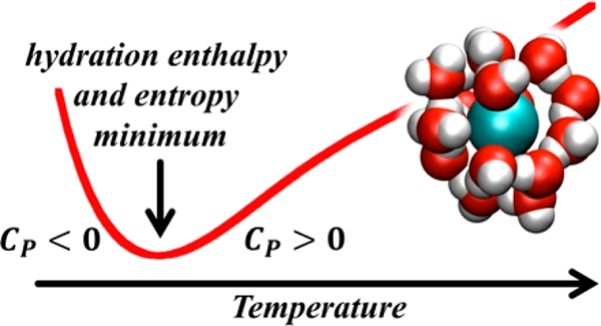

Using simulations and theory, we examine the enthalpy and entropy of hydrophobic hydration which exhibit minima in supercooled water, contrasting with the monotonically increasing temperature dependence traditionally ascribed to these properties. The enthalpy/entropy minima are marked by a negative to positive sign change in the heat capacity at a size-dependent reversal temperature. A Gaussian fluctuation theory accurately captures the reversal temperature, tracing it to water’s distinct thermal expansivity and compressibility influenced by its metastable liquid–liquid critical point.

The insolubility of nonpolar species in aqueous solution underlies a range of biological assembly phenomena, from the formation of lipid membranes to the folding of proteins to the stabilization of hierarchical supramolecular complexes. As such, the hydrophobic effect, which is the tendency of nonpolar substances to aggregate in water driven by their insolubility, has been the subject of scientific scrutiny not only to understand biological function but also to harness hydrophobic assembly phenomena to engineer synthetic nanotechnologies that mimic biology. The simplest solutes studied to gain insight into the origins of the insolubility of nonpolar species in water are the noble gases and alkanes, which benefit from being purely nonpolar so that analyses are not complicated by the presence of ionic or polar contributions to their hydration.

The distribution of a solute (A) between water (w) and an ideal gas (ig) phase is governed by the Ostwald solubility
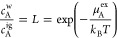
1where *c*_A_^i^ is the concentration of A in phase i (= w or ig), *L* is the Ostwald partition coefficient, *k*_B_*T* is the product of Boltzmann’s constant and the temperature, and μ_A_^ex^ is the excess chemical potential or free energy associated with placing the solute within the liquid. In addition to μ_A_^ex^ being large and positive for nonpolar species indicative of their poor solubility in water, the dissolution of simple nonpolar gases is marked by a number of characteristic thermodynamic signatures associated with hydrophobic hydration:^1,2^ (1) the hydration enthalpy (*h*_A_^ex^ = −*T*^2^ ∂(μ_A_^ex^/*T*)/*∂T*|_*P*_) at room temperature is negative, favoring dissolution; (2) the hydration entropy (*S*_A_^ex^ = −∂μ_A_^ex^/*∂T*|_*P*_) at room temperature is more significantly negative, dominating the positive hydration free energy; and (3) the entropy and enthalpy are increasing functions that are expected to reverse roles and change sign at elevated temperature, indicative of a large positive hydration heat capacity increment (*c*_A_^ex^ = *∂h*_A_^ex^/*∂T*|_*P*_ = *T∂s*_A_^ex^/∂*T*|_*P*_). These thermodynamic signatures have long been attributed to nonpolar solutes inducing a clathrate-like ordering of waters in their hydration shell,^1^ although this is a subject of ongoing debate.^3−5^ To gain a deeper understanding of the role of water structure on moderating nonpolar solute hydration, studies have pushed into the metastable supercooled water regime (*T* < 0 °C) where liquid water presents a more pristine hydrogen bond network order as its density decreases below the temperature of maximum density at 4 °C. Surprisingly, Souda experimentally found that butane and hexane can be taken up and incorporated into amorphous solid water glasses.^6^ Paschek subsequently demonstrated from simulations that the hydration heat capacity of water changes sign from positive to negative values in supercooled water at atmospheric pressure, giving rise to minima in the hydration enthalpy and entropy.^7^ Galamba similarly observed a sign reversal in the hydration heat capacity increment from simulations of alkanes spanning ethane to tetracontane in supercooled water.^8^ Notably, Galamba found that the hydration enthalpy minimum shifted from −40 to −20 °C from ethane to tetracontane, indicating a solute size dependence for this effect. An earlier report by Dill and co-workers hinted at the possibility of sign reversal of the hydration heat capacity at lower temperatures for a two-dimensional description of water, although this was not elaborated on.^9^ Paschek surmised that, in the supercooled regime, argon hydration shell waters more closely resembled the bulk low-density liquid, minimizing the hydration entropy penalty. Galamba, on the other hand, noted that the enthalpy/entropy minimum nearly coincides with the temperature at which the difference in tetrahedral order between low-density and high-density water is greatest, suggesting that this behavior arises from the behavior of supercooled water itself. These observations merit deeper study to clarify the origin of the reversal of the temperature dependence of hydrophobic hydration and its relationship to the properties of supercooled water. Here we report a molecular simulation and statistical thermodynamic analysis in terms of water’s equation-of-state that provides an alternate perspective on the origins of the reversal of the temperature dependence of hydrophobic hydration in supercooled water.

We performed molecular simulations of simple gas hydration in water from deeply supercooled temperatures to the normal boiling point. Isothermal–isobaric ensemble simulations were conducted using GROMACS.^10^ Water was simulated using the TIP4P/2005 force field^11^ over the temperature range of −65 to 100 °C in 5 °C increments at 1 atm pressure. Structural and dynamic properties of the solvent indicate that it remains a liquid over the simulation time even in the supercooled regime. Solute excess chemical potentials were evaluated using Widom test particle insertion.^12,13^ The solutes considered were helium (He), neon (Ne), argon (Ar), krypton (Kr), xenon (Xe), and methane (Me), modeled using Lennard-Jones (LJ) potentials optimized to aqueous solubilities^14^ along with their repulsive Weeks–Chandler–Andersen (WCA) cores.^15^ In addition, the solubilities of hard sphere (HS) solutes with radii *R* (indicating the solvent-excluded size) of up to 3.6 Å were evaluated. Further computational details are provided in the Supporting Information.

The excess chemical potential, enthalpy, and entropy multiplied by the temperature (*Ts*_A_^ex^) of Ar in water are reported in Figure 1a. The simulation enthalpy and entropy were determined by numerically evaluating the derivatives of the chemical potential. At room temperature (25 °C), the predicted thermodynamics of Ar dissolution are representative of the signatures of simple nonpolar solute hydration enumerated above. These signatures continue down to at least −30 °C but diverge from expectations with further decreases in temperature. Notably, the hydration enthalpy exhibits a minimum near −40 °C, below which the enthalpy increases with decreasing temperature. Moreover, the rate of increase in the enthalpy is such that it may be anticipated to become positive just below −60 °C, at which point Ar’s solubility is a maximum. These results indicate that the heat capacity must change sign from positive to even more dramatically negative values as the temperature decreases. The temperature at which the enthalpy is a minimum corresponds to the point at which the heat capacity is zero, denoted as *T*_h_^min^. The interrelationship between the enthalpy and entropy through the heat capacity dictates that the entropy exhibits a minimum at the exact same temperature as the enthalpy (i.e., when *c*_A_^ex^ = 0, it follows that *∂h*_A_^ex^/*∂T*|_*P*_ = 0 and *∂s*_A_^ex^/*∂T*|_*P*_ = 0), although multiplication by the temperature results in the minimum in *Ts*_A_^ex^ occurring at a temperature slightly higher than *T*_h_^min^ (Figure 1a). These observations are not unique to Ar but are also found for noble gases He, Ne, Kr, and Xe as well as Me in water (Figures S1–S5 in the Supporting Information).

**Figure 1:**
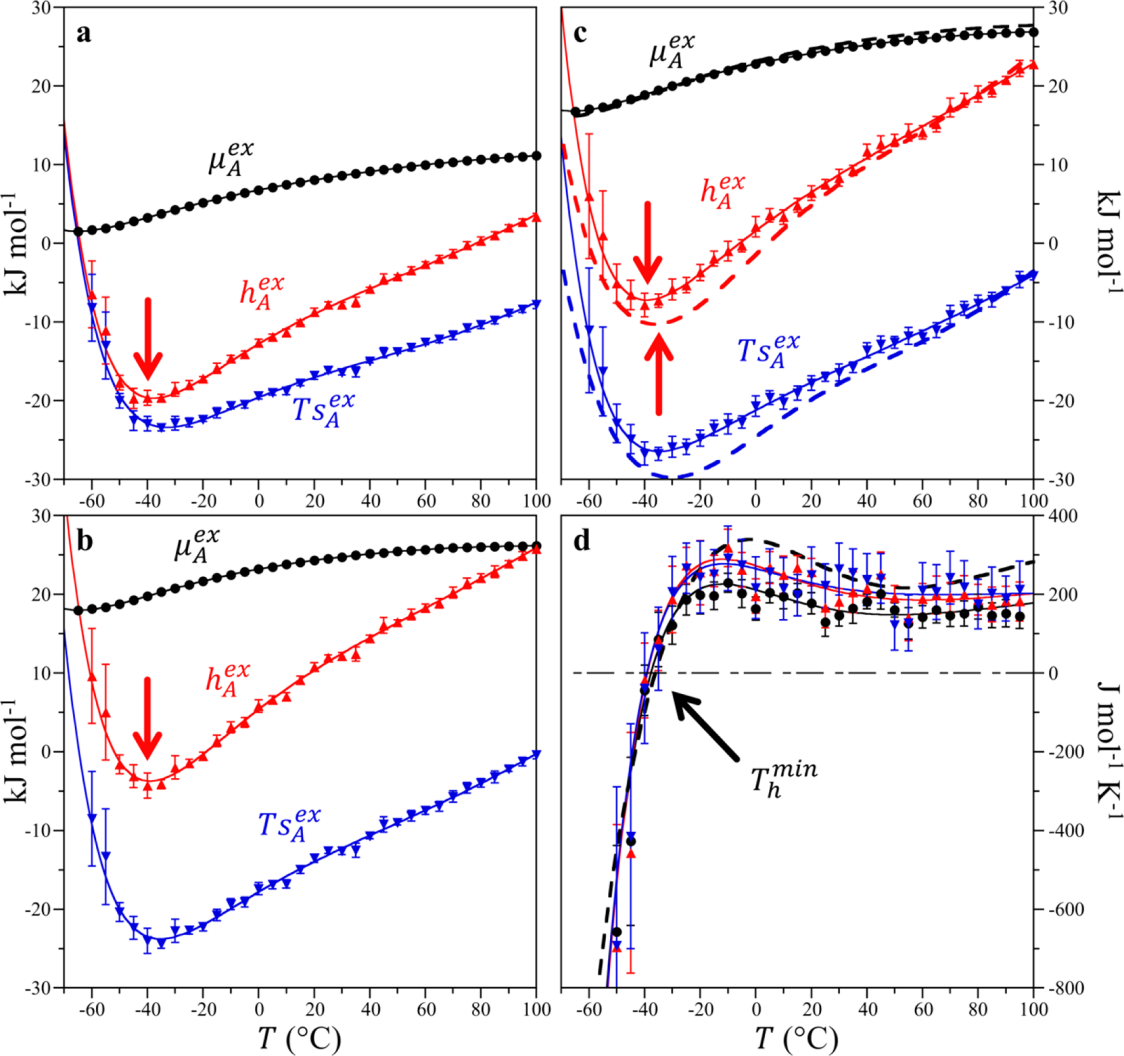
Thermodynamics of Ar-like solute hydration at 1 atm pressure as a function of temperature from −65 to 100 °C. Panels a–c report the excess chemical potential (μ_A_^ex^), enthalpy (*h*_A_^ex^), and entropy (*Ts*_A_^ex^) for an LJ (a), WCA (b), and *R* = 3.2 Å HS solute (c) representation of Ar, respectively.Chemical potential (●), enthalpy (red ▲), and temperature times entropy (blue ▼). The solid lines (—) in a–c correspond to fits of a fifth-order polynomial to the simulation chemical potential (μ_A_^ex^ = ∑_*i*=0_^5^*a*_*i*_(*T*_0_/*T* – 1)^*i*^, where *T*_0_ = 298.15 K is a reference temperature) and taking appropriate temperature derivatives to obtain enthalpy and entropy. The red arrows indicate the enthalpy minima. Panel d reports the simulation heat capacities obtained by numerical differentiation of the enthalpy for the LJ (●), WCA (▲), and HS (blue ▼) representations of Ar. The solid black, red, and blue lines in d correspond the LJ, WCA, and HS heat capacities determined from the fits in a–c, while the thin long–short dashed horizontal line (-−) indicates a heat capacity of zero. The thick dashed lines (--, blue --, and red --) in c and d correspond to the predictions of IGFT for an *R* = 3.2 Å HS solute. The error bars indicate one standard deviation. The errors in the chemical potentials are smaller than the symbols.

To gain insight into the origins of the enthalpy minimum, we first consider the role of solute–water attractive interactions on Ar’s hydration thermodynamics. Figure 1b,c reports the thermodynamics of hydrating a repulsive WCA Ar and a 3.2 Å radius HS solute, approximately the size of Ar. The hydration free energies of the WCA and HS solutes are greater than those of the LJ representation of Ar as a result of the neglected attractive interactions, reflected in the substantive increase in their hydration enthalpies. The hydration entropies of these solutes, on the other hand, are to a first approximation the same as that of the LJ Ar. Most importantly, however, the enthalpies and entropies of all of these solutes exhibit minima in the vicinity of −40 °C. The agreement between *T*_h_^min^ for all of these representations of Ar can be seen from their heat capacities evaluated by numerical differentiation of the simulation results (Figure 1d). Above the melting point of water, the heat capacities are positive with values in reasonable agreement with the mean experimental value of 177 J/(mol K).^16^ While the errors in the calculated heat capacity increase with decreasing temperature, the sign of the heat capacities for all of these solutes cross zero and change sign over a narrow range of temperatures near −40 °C. A more accurate estimate of *T*_h_^min^ can be determined by fitting the simulation free energies to the fifth-order polynomial μ_A_^ex^ = ∑_*i*=0_^5^*a*_*i*_(*T*_0_/*T* – 1)^*i*^, where *T*_0_ = 298.15 K is a reference temperature and *a*_*i*_ represents parameters fitted to simulation. The accuracy of this polynomial can be verified by comparison against our simulation results for the excess chemical potential, enthalpy, entropy, and heat capacity for the Ar-like solutes in Figure 1.

The solute size dependence for *T*_h_^min^ determined from the polynomial fits to our simulation hydration free energies is reported in Figure 2 for the HS, LJ, and WCA solutes. For HS radii smaller than that of a water molecule (*R* < *d*_ww_/2 ≈ 1.4 Å, where *d*_ww_ is water’s effective diameter), *T*_h_^min^ is practically independent of solute size and equal to −50 °C within the simulation error. Beyond this radius, *T*_h_^min^ systematically increases from −50 to −30 °C for the largest solutes considered. No difference among the LJ, WCA, and HS enthalpy minimum temperatures is observed within the simulation error bars when compared on the basis of the solute size, indicating that attractive solute–water interactions play almost no role in this effect. The increase in *T*_h_^min^ with solute size is in qualitative agreement with the shift reported by Galamba for alkanes of increasing length.^8^ We note that the solute size dependence indicated in Figure 2 is significant for the potential experimental validation of the predicted enthalpy/entropy minimum. Specifically, while *T*_h_^min^ for the smallest solutes falls below the homogeneous nucleation temperature of ice (−38 °C),^17^ our results along with those reported by Galamba^8^ suggest that *T*_h_^min^ can rise above this temperature for reasonably sized solutes.

**Figure 2:**
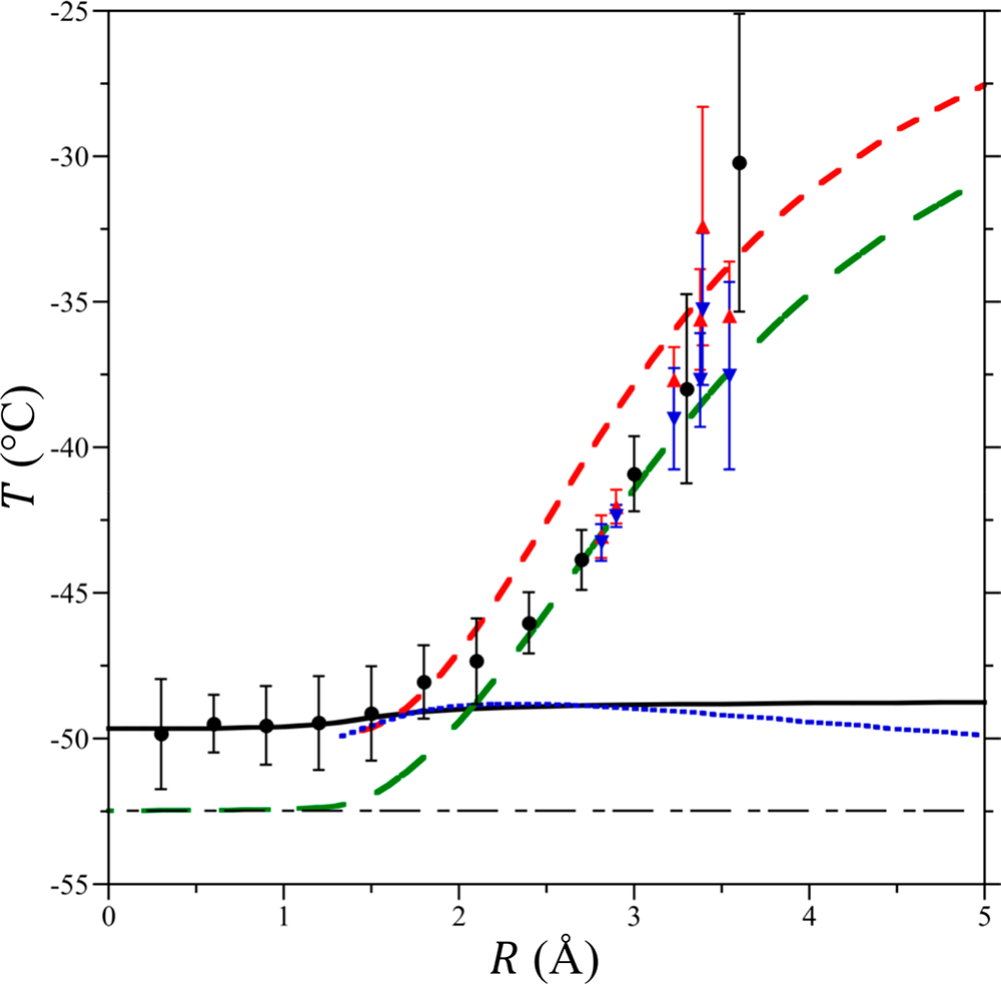
Size dependence of the hydration enthalpy minimum temperature at 1 atm. The minimum in the simulation results was determined from the polynomial fits of the excess chemical potentials. The solvent-excluded radii of the LJ and WCA solutes were evaluated from their thermal radii as described in the Supporting Information. HS solutes (●), LJ solutes (red ▲), and WCA solutes (blue ▼). Scaled particle theory prediction assuming a hard sphere water diameter of 2.7 Å (thick solid black line **–**),^18^ IGFT prediction (thick red medium dashed line --), IGFT prediction assuming a constant compressibility (*κ*_*T*_ = 5 × 10^–5^ atm^–1^) (thick short dashed blue line --); minimum in α + ∂ ln χ/*∂T*|_*P*_ (thick green long dashed line **––**), and minimum in α (-−). The error bars indicate one standard deviation.

Scaled particle theory provides insight into the origin of the *T*_h_^min^ plateau for *R* < *d*_ww_/2.^18^ For HS solutes, the excess chemical potential is determined from the probability of observing a solute-sized cavity in water devoid of water oxygens, *p*_0_/(*R*). For spherical observation volumes so small that at most one water oxygen could be observed within its boundary, the excess chemical potential of a solute of that size is exactly
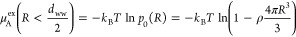
2where ρ is the solvent number density. *T*_h_^min^ values obtained by utilizing the simulation water densities in eq 2 are in excellent agreement with those obtained from particle insertion (Figure 2; see the Supporting Information for fittings of simulated water’s equation-of-state properties used in our theoretical expressions). In the limit of a solute of zero size (*R* = 0), eq 2 predicts that *T*_h_^min^ is observed when ∂^2^ρ/*∂T*^2^|_*P*_ = 2*ρα*/*T*, where α = −∂ ln ρ/*∂T*|_*P*_ is the solvent’s thermal expansion coefficient. (See the Supporting Information for the derivation.) Given that α is typically a small number, when α is divided by the temperature, ∂^2^ρ/*∂T*^2^|_*P*_ is expected to be nearly zero at *T*_h_^min^, suggesting that the enthalpy minimum is almost coincident with a minimum in α. While for most liquids α is a positive, monotonically varying function of temperature, α for water famously becomes negative below its temperature of maximum density. The decrease in α continues well into the supercooled regime until −52.5 °C for TIP4P/2005 water (Figure 3a), below which α increases with further decreases in temperature. This minimum in α in the supercooled regime has also been inferred from experiments of D_2_O trapped within mesoporous silica.^19^ Simulation results for *T*_h_^min^ for small solutes are in reasonable agreement with the temperature at which α is a minimum (Figure 2), greater by only a couple of degrees. While eq 2 predicts a size dependence for *T*_h_^min^ (see the Supporting Information), it is only a weakly varying function of the solute radius, yielding an essentially flat plateau for *R* < *d*_ww_/2. In addition, we include scaled particle theory predictions for *T*_h_^min^ for solutes larger than *d*_ww_/2 in Figure 2. While scaled particle theory has been applied to understand a range of hydrophobic hydration phenomena, this theory predicts only an ∼1 °C change in *T*_h_^min^ from point-like to infinitely sized solutes.

**Figure 3:**
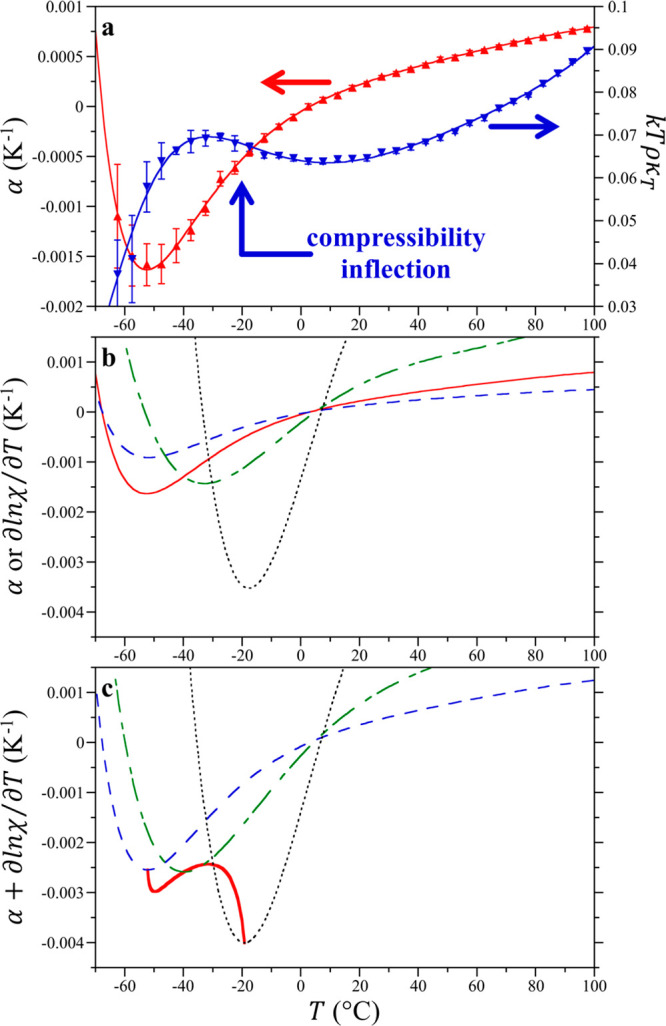
Equation-of-state components of the IGFT model of hydrophobic hydration. (a) Thermal expansivity and normalized compressibility of TIP4P/2005 water from −65 to 100 °C at 1 atm. α (red ▲) and *k*_B_*Tρκ*_*T*_ (blue ▼). The solid lines indicate fits detailed in the Supporting Information. The arrows indicate the axes to which each data set of corresponds. The simulation error bars report one standard deviation. (b) α and ∂ ln χ/*∂T*|_*P*_ as a function of temperature utilized by the IGFT expression of the enthalpy (eq 5). α (thin red −); (thin medium dashed blue line --) ∂ ln χ/*∂T*|_*P*_ for an *R* = 1.4 Å HS solute; (thin long–short dashed green line -−) ∂ ln χ/*∂T*|_*P*_ for an *R* = 3.2 Å HS solute, and ∂ ln χ/*∂T*|_*P*_ for an *R* = ∞ HS solute (--). (c) Sum α + ∂ ln χ/*∂T*|_*P*_ controls the enthalpy minimum temperature. (Thin medium dashed blue line --) sum for an *R* = 1.4 Å HS solute, (thin long–short dashed green line −-) sum for an *R* = 3.2 Å HS solute; sum for an *R* = ∞ HS solute (--), and (thick solid red line **–**) trace of minima in α + ∂ ln χ/*∂T*|_*P*_ for solutes of *R* = 1.4 Å to ∞.

For solutes larger than *d*_ww_/2, multibody correlations between water molecules must be accounted for when evaluating the chemical potential. In the case of information limited to water pair correlations embodied in the water oxygen–oxygen radial distribution function, information theory predicts that solvent occupation fluctuations within solute-sized observation volumes are best described by a discrete Gaussian form.^20^ Assuming that the discrete Gaussian can be approximated using a continuous function, the excess chemical potential of an HS solute in water is
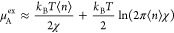
3where ⟨*n*⟩ = ρ(4*πR*^3^/3) is the average number of waters residing within an observation sphere determined by the product of the solvent number density and volume, while χ = (⟨*n*^2^ ⟩ – ⟨*n*⟩^2^/⟨*n*⟩ is the normalized solvent fluctuation in the observation sphere. While χ is determined by an integral over water’s radial distribution function performed over the observation volume, Ashbaugh, Vats, and Garde^21^ recently developed an analytical expression for χ in spherical volumes that smoothly interpolates between the known microscopic and macroscopic limits to provide an excellent quantitative approximation away from the liquid–vapor critical point over all observation volume radii
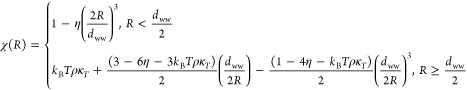
4In this expression, *κ*_*T*_ is the macroscopic solvent isothermal compressibility and η = *πρd*_ww_^3^/6 is the solvent packing fraction. The effective solvent diameter fitted to our simulation results is *d*_ww_ = 2.663 Å, closely corresponding to the separation between water oxygens at which the radial distribution function first crosses one.^21^ This expression enables eq 3 to predict the hydration free energies of HS solutes. While eq 4 interpolates the normalized cavity occupation fluctuations over all radii, the range of solute sizes for which eq 3 is applicable is limited due to its divergence below *d*_ww_/2, where the continuous distribution approximation breaks down and the necessity of considering higher-order moments of the distribution for larger solutes where interfacial contributions become important. Nevertheless, eq 3 is accurate for atomically sized volumes in water, the size range of interest here. Collectively, eqs 3 and 4 are referred to as interpolated Gaussian fluctuation theory (IGFT).

IGFT accurately predicts the chemical potential of the 3.2 Å HS solute over the entire range of temperatures simulated and provides an excellent prediction of the solute’s hydration enthalpy, entropy, and heat capacity (Figure 1c,d). Most importantly, IGFT accurately predicts the observation of a minimum in the hydration enthalpy and the corresponding change in sign of the hydration heat capacity close to that determined from simulation. This theory performs similarly well when applied to a range of HS solute radii from 1.5 to 3.5 Å (Figures S6–S10 in the Supporting Information), lending confidence to the accuracy of this theory applied to simple nonpolar solutes.

The solute size dependence of *T*_h_^min^ is semiquantitatively captured by IGFT (Figure 2), demonstrating the necessity of accounting for solvent density fluctuations when describing hydration in the supercooled regime as captured through water’s compressibility. Above the melting point, water’s compressibility has previously been assumed to be nearly independent of temperature, simplifying information theory predictions for the functional dependence of the solute chemical potential on the equation-of-state properties of water. In the supercooled regime, however, the compressibility exhibits a maximum as a function of temperature that can be traced to the Widom line emanating from water’s metastable second liquid–liquid critical point (@ −101 °C and 1837 atm for TIP4P/2005 water^22^). This compressibility maximum is reflected in the maximum in *k*_B_*TρκT* near −35 °C in TIP4P/2005 water (Figure 3a). If a constant value of *κ*_*T*_ = 5 × 10^–5^ atm^–1^ is assumed, the mean compressibility of liquid water is between 0 and 100 °C, IGFT incorrectly predicts a nearly size-independent *T*_h_^min^ coincident with the small solute size plateau and is in close agreement with the predictions of scaled particle theory (Figure 2). It may thereby be hypothesized that the peculiar expansive and compressive equation-of-state properties of supercooled water play a role in the observed size dependence of *T*_h_^min^. Notably, scaled particle theory also neglects the solvent compressibility in its predictions at constant pressure.

The connection between the enthalpy minimum and water’s equation-of-state can be made more explicit following IGFT. From the temperature derivative of eq 3, the enthalpy is
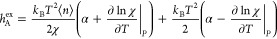
5The first and second terms on the right-hand side of this expression follow from the corresponding first and second terms in eq 3. While the second term is not negligible, the magnitude of the enthalpy (and chemical potential) is dominated by the first term of eq 5 (or eq 3 for μ_A_^ex^). Given that the prefactor of this term, essentially corresponding to the chemical potential multiplied by the temperature, is a monotonically increasing function of temperature, the enthalpy minimum is controlled by the sum within the parentheses, α + ∂ln χ/*∂T*|_*P*_. Given that α is solely a solvent property, the solute size dependence of *T*_h_^min^ follows from the size dependence of ∂ln χ/*∂T*|_*P*_ as dictated by eq 4 (Figure 3b). For a solute comparable in size to the solvent radius (*d*_ww_/2*R* ≈ 1), the compressibility contributions cancel and χ effectively depends only on the solvent density. As a result, the minimum in ∂ ln χ/*∂T*|_*P*_ nearly coincides with the minimum in α. On the other hand, χ is equal to *k*_B_*Tρκ*_*T*_ for a solute of infinite size (*d*_ww_/2*R* = 0) so that the minimum in ∂ln χ/*∂T*|_*P*_ is dictated by the inflection point in *k*_B_*Tρκ*_*T*_ at −20 °C (Figure 3a), between the positive concavity observed above water’s melting point and the negative concavity in the supercooled region where *k*_B_*Tρκ*_*T*_ exhibits a maximum. For solutes of intermediate size, the minimum in ∂ln χ/*∂T*|_*P*_ falls between these two extremes as dictated by eq 4 (Figure 3b). Subsequently, the minimum in the sum α + ∂ln χ/*∂T*|_*P*_ systematically increases with increasing solute size from the minimum in α temperature to a plateau for infinitely sized solutes, although this plateau falls outside the range of solute sizes for which the Gaussian fluctuation approximation applies. We find excellent agreement when comparing the minima in α + ∂ln χ/*∂T*|_*P*_ temperatures (Figure 3c) against the *T*_h_^min^ determined from IGFT (Figure 2), with α + ∂ln χ/*∂T*|_*P*_ underpredicting *T*_h_^min^ by only ∼2 °C. Despite the expected failure of eq 3 for solutes of decreasing size, the minimum in α + ∂ln χ/*∂T*|_*P*_ predicts a small solute plateau when applied below *d*_ww_/2 that coincides with the minimum in α, in reasonable agreement with the *T*_h_^min^ from simulation (Figure 2). This comparison supports the hypothesis that the enthalpy minimum in the supercooled region, below which the temperature dependence of hydrophobic hydration changes sign, is tied to the interplay between the equation-of-state properties α and *k*_B_*Tρκ*_*T*_ as moderated through eq 4 following IGFT.

In conclusion, we have demonstrated that the classic signatures of hydrophobic hydration are not inviolable but can exhibit a significant negative heat capacity increment in the supercooled regime to reverse the supposed characteristic temperature dependence of the hydration enthalpy and entropy. While it has been previously proposed that this reversal is a result of the nonpolar solutes breaking down the increasingly perfected structure of supercooled low-density water,^7^ our analysis suggests that this behavior is attributable to the peculiar equation-of-state properties of liquid water in this regime. For solutes so small that they can interact only with a single water molecule, no enthalpy minimum would be expected if water structure breaking played a role, but simulations indicate a *T*_h_^min^ for small solutes that is attributable to the occurrence of a minimum in water’s thermal expansivity at −52.5 °C. For larger solutes, IGFT accurately predicts that *T*_h_^min^ increases with increasing solute size from point-like solutes (*R* = *d*_ww_/2) to those approximately 3.6 Å in radius. The main inputs into IGFT are water’s density and compressibility. Water’s structure enters only through the effective diameter of water, assumed here to be temperature-independent, that dictates the point at which pair correlations begin to contribute to density fluctuations within a spherical observation cavity and does not invoke liquid water’s three-dimensional hydrogen-bonded network. Within the context of IGFT, water’s ramified structure contributes only through its impact on its equation-of-state, which is not insignificant in supercooled water. The solute size dependence of *T*_h_^min^ results from the interplay between the minimum in α and the inflection point between the maximum in *k*_B_*Tρκ*_*T*_ at supercooled temperatures and its minimum above the melting point of water. The inflection in *k*_B_*Tρκ*_*T*_ is tied to the maximum in *κ*_*T*_, which can be traced to the metastable second liquid–liquid critical point found from simulations of TIP4P/2005 water^22^ and strongly believed for real water based on current evidence.^23,24^ As such, IGFT marks the change in the sign of the hydration heat capacity increment with respect to the unique equation-of-state properties of liquid water in the supercooled regime. This is not to say that atomic nonpolar solutes do not disrupt low-density water’s hydrogen bond network under supercooled conditions. Rather, these structural imperfections accompany the normal Gaussian fluctuations that open suitably sized cavities in which the solute could reside. Extrapolation of the hydration enthalpies of the simple gases (Figure 1a and Figures S1–S5 in the Supporting Information) to temperatures below −60 °C suggests that the enthalpy will cross zero to become positive, spurred by their dramatically negative heat capacities (e.g., Figure 1d). Below this temperature, their solubilities will become increasingly meager. As a result, the diminishing solubility of nonpolar moieties could promote the refolding of cold denatured proteins as was recently described from simulations in supercooled water below −70 °C.^25^

## Supporting Information Available

Details on the molecular simulations performed; simulation results for LJ gases He, Ne, Kr, Xe, and Me, and HS solutes with radii 1.5, 2, 2.5, 3, and 3.5 Å; details on the fits of water’s equation of state to polynomial functions; and the analysis of subpoint-like solute hydration using scaled-particle theory (PDF)
