# Supplementary material for: Reversal of the Temperature Dependence of Hydrophobic Hydration in Supercooled Water
Source: J Phys Chem Lett. 2021 Aug 26;12(34):8370–5. doi: 10.1021/acs.jpclett.1c02399 (PMC8419862; doi:10.1021/acs.jpclett.1c02399)
Supplement: Supplementary file 1 — jz1c02399_si_001.pdf [file jz1c02399_si_001.pdf]

Supporting Information for

Reversal of the Temperature Dependence of Hydrophobic Hydration in Supercooled Water

Henry S. Ashbaugh*

Department of Chemical and Biomolecular Engineering, Tulane University, New Orleans, LA 70118

In this document we provide additional details on: the molecular simulations performed; figures regarding the hydration thermodynamics of the gases helium, neon, krypton, xenon, and methane; figures regarding the hydration thermodynamics of hard spheres of radius 1.5 Å to 3.5 Å; details of the fitting of water's equation-of-state properties to a polynomial function; and a detailed derivation of the hydration enthalpy minimum criterion of sub-point like solutes established by scaled-particle theory.

Molecular simulation details. Molecular dynamics simulations of pure water were performed using GROMACS 5.1 [1]. Water was modeled using the TIP4P/2005 force field [2] which accurately captures water's liquid equation-of-state properties. A total of 909 water molecules were included in the cubic simulation box. Non-bonded Lennard-Jones interactions were truncated beyond a separation of 9 Å with a mean-field dispersion correction for longer-range contributions to the energy and pressure. Electrostatic interactions were evaluated using the particle mesh Ewald Summation method with a real space cutoff of 9 Å [3]. Simulations of liquid water were performed from temperatures ranging from -65°C to 100°C in 5 K increments at a pressure of 1 atm for a total of 33 state points. The temperature and pressure were regulated using the Nosé-Hoover [4, 5]

* corresponding author. Email: hanka@tulane.edu.

thermostat and Parrinello-Rahman barostat [6], respectively. Water was held rigid using the SETTLE algorithm [7]. Following 5 ns of equilibration, each state point was simulated for 200 ns for evaluation of equilibrium averages. The equations of motion were integrated using a time step of 2 fs. Simulation configurations were saved every 1 ps (200,000 total configurations at each state point) for post-simulation analysis of solute chemical potentials.

The excess chemical potential describes the contribution to the solute chemical potential arising from interactions between the solute and water. Assuming the solute is at equilibrium between an ideal gas and solution at infinite dilution, the excess chemical potential was determined from simulations using Widom's test particle insertion formula [8] extended to the isothermal-isobaric ensemble [9]

$$\mu_A^{ex} = - k_B T ln \frac{\langle V exp(- \Delta\varphi_{aw}/k_B T)\rangle}{\langle V \rangle}, \tag{S1}$$

where V is the system volume, $\Delta\varphi_{Aw}$ is the interaction energy of the randomly inserted solute (A) with water (w), and the brackets $\langle ... \rangle$ indicate averages evaluated from pure solvent configurations. 12,500 random test particle insertions were attempted for each configuration, for a total of 2.5×10^9 insertions per state point. The solutes considered were helium, neon, argon, krypton, xenon, and methane modeled using Lennard-Jones (LJ) interactions previously optimized to reproduce their experimental solubility in liquid water above the melting point [10]. In addition to LJ representations, the free energies for hydrating its Weeks-Chandler-Andersen (WCA) repulsive core were also evaluated [11]

$$\varphi_{\mathrm{WCA}}(r) = \begin{cases} \varphi_{\mathrm{LJ}}(r) + \varepsilon & r \leq 2^{1/6}\sigma \\ 0 & r > 2^{1/6}\sigma \end{cases}, \tag{S2}$$

where σ and ε are the LJ diameter and well-depth, respectively.

To compare the simulation results for LJ, WCA, and HS solutes we need to define their effective sizes. For the HS solutes we simply use their solvent excluded radius, R, which is identified as the distance of closest approach between the solute center and a water oxygen. In the case of the LJ and WCA solutes that poses softer cores than the HS solutes we use their thermal radii. The thermal radius, R_{th}, is determined by the distance at which the repulsive core of the solute-water interaction is equal to the thermal energy, $\varphi_{\mathrm{WCA}}(R_{th}) = k_B T$. For the both the LJ and WCA solutes this is

$$R_{th} = \sigma\left[\frac{2}{1 + (k_B T/\varepsilon)^{1/2}}\right]^{1/6}. \tag{S3}$$

We subsequently equate the solvent-excluded and thermal radii in Figure 2, $R_{th} = R$, which is an excellent approximation.

Molecular simulation results. Figures S1 – S5 report the hydration chemical potentials, enthalpies, and entropies for helium (He), neon (Ne), krypton (Kr), xenon (Xe), and methane (Me) determined from simulation. Figures S6 – S10 report the hydration chemical potentials, enthalpies, and entropies for hard sphere solutes with radii of 1.5 Å, 2 Å, 2.5 Å, 3 Å, and 3.5 Å determined from simulation.

Fits of water's equation-of-state results. To analyze the temperature derivatives of the water's equation-of-state properties used in the scaled-particle theory (eq. (2)) and interpolated Gaussian fluctuation theory (eqs. (3) – (5)) expressions, we performed least squares fits of ρ and $k_B T \rho \kappa_T$ to the sixth order polynomial

$$\Gamma = \sum_{i=0}^{6} \gamma_i \left(\frac{T_0}{T} - 1\right)^i, \tag{S4}$$

where Γ is the property of interest, T_0 = 298.15K is a reference temperature, and the γ_i are parameters fitted to simulation results. While the free energies from simulation were fitted to an

analogous fifth order polynomial, we obtained a more accurate representation of the equation-of-state results using a sixth order polynomial. While the fit of $k_B T \rho \kappa_T$ is reported in Figure 3a, the fit for ρ is reported in Figure S11. The thermal expansion coefficient $\alpha = -\partial ln\rho/\partial T|_P$ which results from the temperature derivative of the density is compared against simulation in Figure 3a.

Scaled-particle theory analysis of the enthalpy minimum for small solutes. In the limit of a solute whose solvent excluded radius is less than half the diameter of a water molecule its excess chemical potential is analytically determined from scaled-particle theory [12] as

$$\mu_{\mathrm{A}}^{ex}(R < d_{ww}/2) = -k_B T ln\left(1 - \rho\frac{4\pi R^3}{3}\right). \tag{S5}$$

In the limit of a cavity of zero volume ($R = 0$) the excess chemical potential is zero along with all the other hydration thermodynamic properties of the solute. The hydration enthalpy for any non-zero volume solute as determined from eq. (S5) could exhibit to minimum as a function of temperature. This limiting behavior can be determined by dividing the hydration free energy by the solute's volume. The corresponding hydration enthalpy normalized by the solute volume is

$$\frac{1}{4\pi R^3/3} h_{\mathrm{A}}^{ex}(R < d_{ww}/2) = -T^2 \frac{\partial \mu_{\mathrm{A}}^{ex}(R)/T}{\partial T}\bigg|_P = -\frac{k_B T^2}{1 - \rho 4\pi R^3/3} \frac{\partial \rho}{\partial T}\bigg|_P. \tag{S6}$$

The volume normalized heat capacity is subsequently

$$\frac{1}{4\pi R^3/3} c_{\mathrm{A}}^{ex}(R < d_{ww}/2) = \frac{\partial h_{\mathrm{A}}^{ex}(R)}{\partial T}\bigg|_P$$

$$= -\frac{2k_B T}{1 - \rho 4\pi R^3/3} \frac{\partial \rho}{\partial T}\bigg|_P - \frac{k_B T^2 4\pi R^3/3}{(1 - \rho 4\pi R^3/3)^2}\left(\frac{\partial \rho}{\partial T}\bigg|_P\right)^2 - \frac{k_B T^2}{1 - \rho 4\pi R^3/3} \frac{\partial^2 \rho}{\partial T^2}\bigg|_P \tag{S7}$$

The minimum in the hydration enthalpy is determined by the temperature at which the heat capacity is zero. For sub-point like solutes, this minimum occurs when the following criterion is satisfied

$$\frac{\partial^2 \rho}{\partial T^2}\bigg|_P = -\frac{2\partial \rho}{T \partial T}\bigg|_P - \frac{4\pi R^3/3}{1 - \rho 4\pi R^3/3}\left(\frac{\partial \rho}{\partial T}\bigg|_P\right)^2. \tag{S8}$$

In the limit of a solute of zero size, this criterion becomes

$$lim_{R\to 0}\frac{\partial^2\rho}{\partial T^2}\bigg|_P = -\frac{2\partial\rho}{T\partial T}\bigg|_P = \frac{2\rho\alpha}{T}. \quad \text{(S9)}$$

So, in the limit of a solute of zero size the minimum in the enthalpy terminates at the temperature when eq. (S9) is satisfied. Above the melting point of water $\partial\rho/\partial T|_P$ is negative and $\partial^2\rho/\partial T^2|_P$ is negative (Figure S11). As a result, eq. (S9) cannot be satisfied above the melting point of water. For supercooled water, however, $\partial\rho/\partial T|_P$ is positive (α is negative) over a significant range of temperatures and it is possible that this criterion could be satisfied. Given that α is small in magnitude and even smaller when divided by the temperature, eq. (S9) suggests the enthalpy minimum will be satisfied when $\partial^2\rho/\partial T^2|_P$ is negative but close to zero. $\partial^2\rho/\partial T^2|_P$ is zero at -52.5°C and the normalized hydration enthalpy minimum for a solute of zero size is at -50°C (Figure 2), demonstrating this intuition is largely correct. Given that $\partial^2\rho/\partial T^2|_P$ is negative above -52.5°C, based on eq. (S9) it is sensible that the actual hydration enthalpy minimum occurs at a slightly higher temperature. The second term in eq. (S8) that depends on the solute radius indicates that $\partial^2\rho/\partial T^2|_P$ will become more negative with increasing solute size. As a result, eq. (S8) suggests the enthalpy minimum temperature will shift to slightly higher temperatures as the solute size increases up the size $R = d_{ww}/2$, after which pair and higher order correlations are needed to be able to predict the enthalpy minimum temperature.

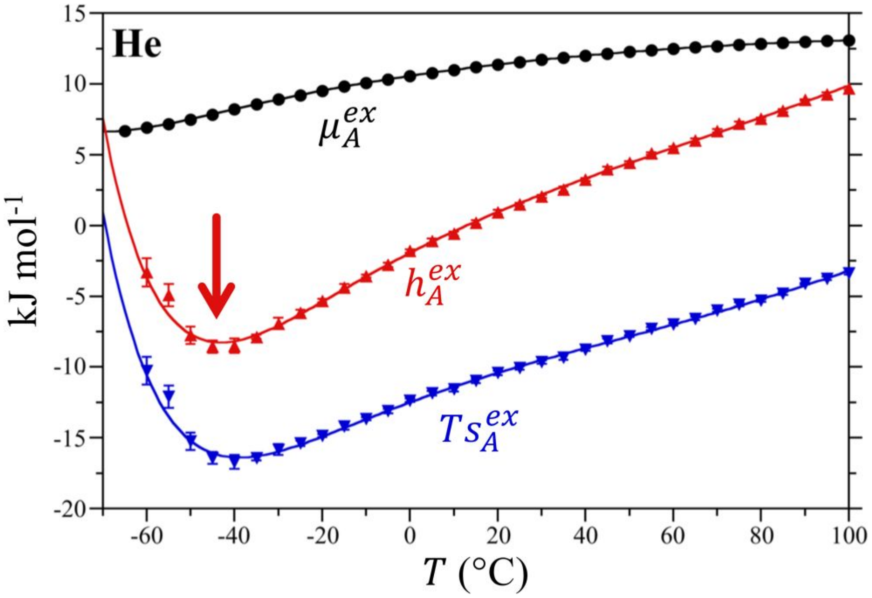

Figure S1. Thermodynamics of helium dissolution in water at 1 atm pressure as a function of temperature from -65°C to 100°C. Helium is modelled using a Lennard-Jones potential. The solid black circles (●), solid red triangles (▲), and solid blue upside-down triangles (▼) correspond the simulation results for the hydration chemical potential, enthalpy, and entropy, respectively. The solid lines (———) correspond to fits of a fifth order polynomial to the simulation chemical potential ($\mu_A^{ex} = \sum_{i=0}^{5} a_i (T_0/T - 1)^i$, where T_0 = 298.15K is a reference temperature) and taking appropriate temperature derivatives to obtain enthalpy and entropy. The red arrow indicates the enthalpy minimum. The simulation error bars correspond to one standard deviation.

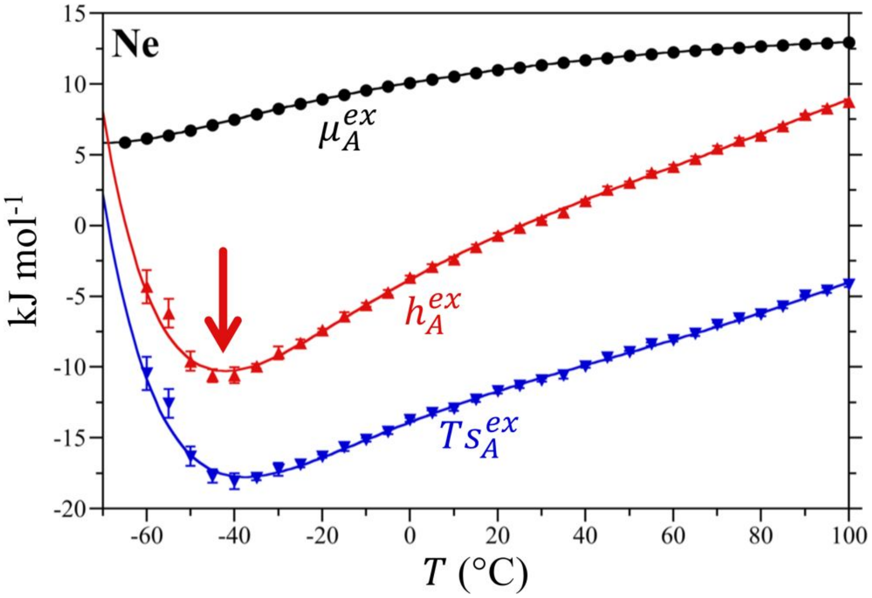

Figure S2. Thermodynamics of neon dissolution in water at 1 atm pressure as a function of temperature from -65°C to 100°C. Neon is modelled using a Lennard-Jones potential. The solid black circles (●), solid red triangles (▲), and solid blue upside-down triangles (▼) correspond the simulation results for the hydration chemical potential, enthalpy, and entropy, respectively. The solid lines (———) correspond to fits of a fifth order polynomial to the simulation chemical potential ($\mu_A^{ex} = \sum_{i=0}^{5} a_i (T_0/T - 1)^i$, where T_0 = 298.15K is a reference temperature) and taking appropriate temperature derivatives to obtain enthalpy and entropy. The red arrow indicates the enthalpy minimum. The simulation error bars correspond to one standard deviation.

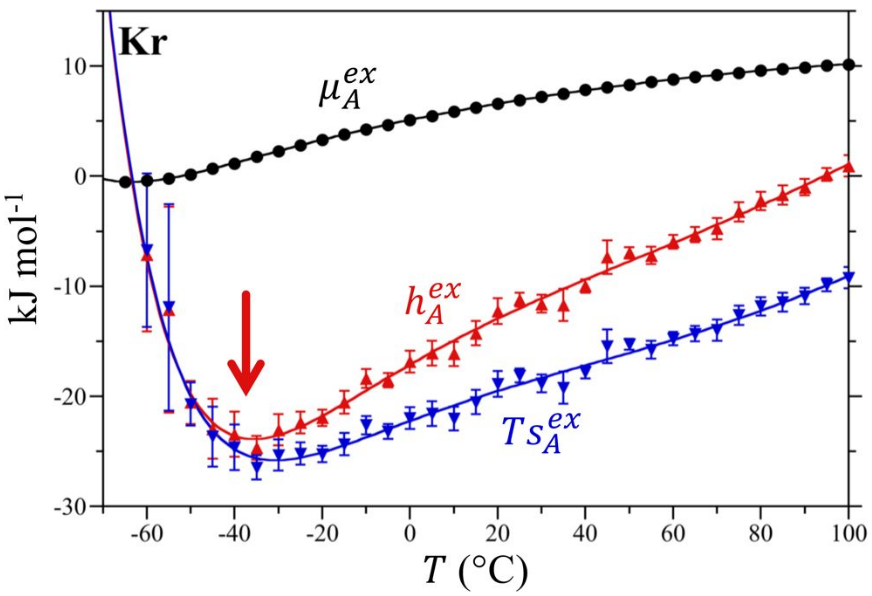

Figure S3. Thermodynamics of Krypton dissolution in water at 1 atm pressure as a function of temperature from -65°C to 100°C. Krypton is modelled using a Lennard-Jones potential. The solid black circles (●), solid red triangles (▲), and solid blue upside-down triangles (▼) correspond the simulation results for the hydration chemical potential, enthalpy, and entropy, respectively. The solid lines (———) correspond to fits of a fifth order polynomial to the simulation chemical potential ($\mu_A^{ex} = \sum_{i=0}^{5} a_i (T_0/T - 1)^i$, where T_0 = 298.15K is a reference temperature) and taking appropriate temperature derivatives to obtain enthalpy and entropy. The red arrow indicates the enthalpy minimum. The simulation error bars correspond to one standard deviation.

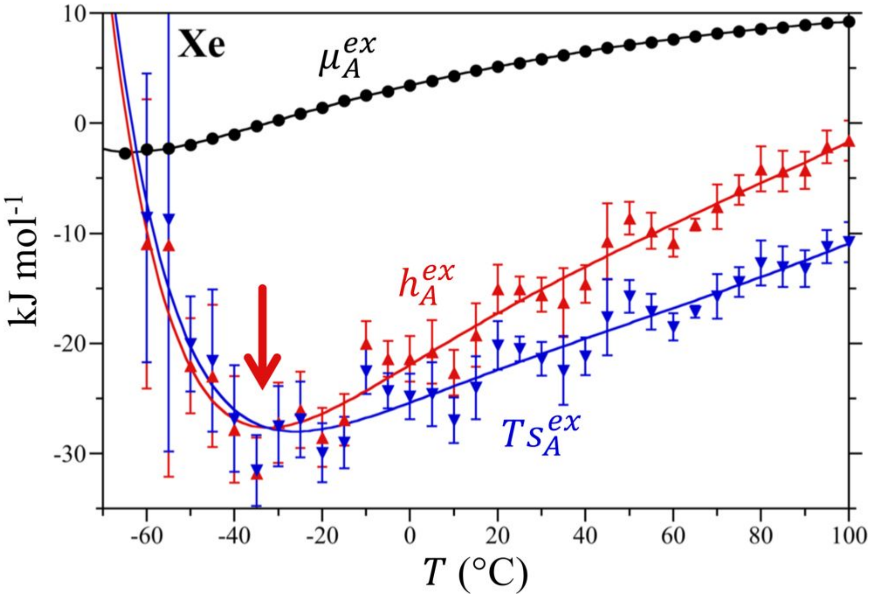

Figure S4. Thermodynamics of xenon dissolution in water at 1 atm pressure as a function of temperature from -65°C to 100°C. Xenon is modelled using a Lennard-Jones potential. The solid black circles (●), solid red triangles (▲), and solid blue upside-down triangles (▼) correspond the simulation results for the hydration chemical potential, enthalpy, and entropy, respectively. The solid lines (———) correspond to fits of a fifth order polynomial to the simulation chemical potential ($\mu_A^{ex} = \sum_{i=0}^{5} a_i (T_0/T - 1)^i$, where T_0 = 298.15K is a reference temperature) and taking appropriate temperature derivatives to obtain enthalpy and entropy. The red arrow indicates the enthalpy minimum. The simulation error bars correspond to one standard deviation.

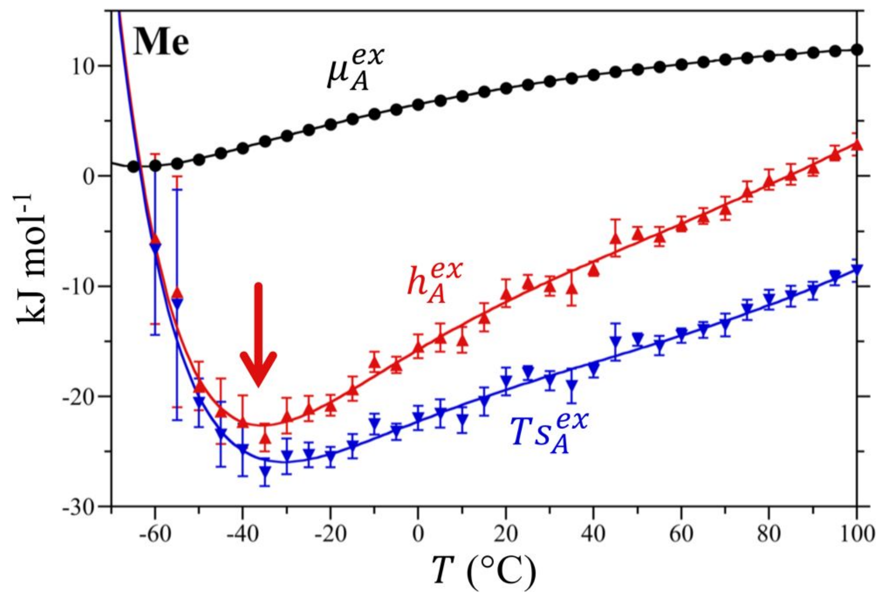

Figure S5. Thermodynamics of methane dissolution in water at 1 atm pressure as a function of temperature from -65°C to 100°C. Methane is modelled using a united atom Lennard-Jones potential. The solid black circles (●), solid red triangles (▲), and solid blue upside-down triangles (▼) correspond the simulation results for the hydration chemical potential, enthalpy, and entropy, respectively. The solid lines (———) correspond to fits of a fifth order polynomial to the simulation chemical potential ($\mu_A^{ex} = \sum_{i=0}^{5} a_i (T_0/T - 1)^i$, where T_0 = 298.15K is a reference temperature) and taking appropriate temperature derivatives to obtain enthalpy and entropy. The red arrow indicates the enthalpy minimum. The simulation error bars correspond to one standard deviation.

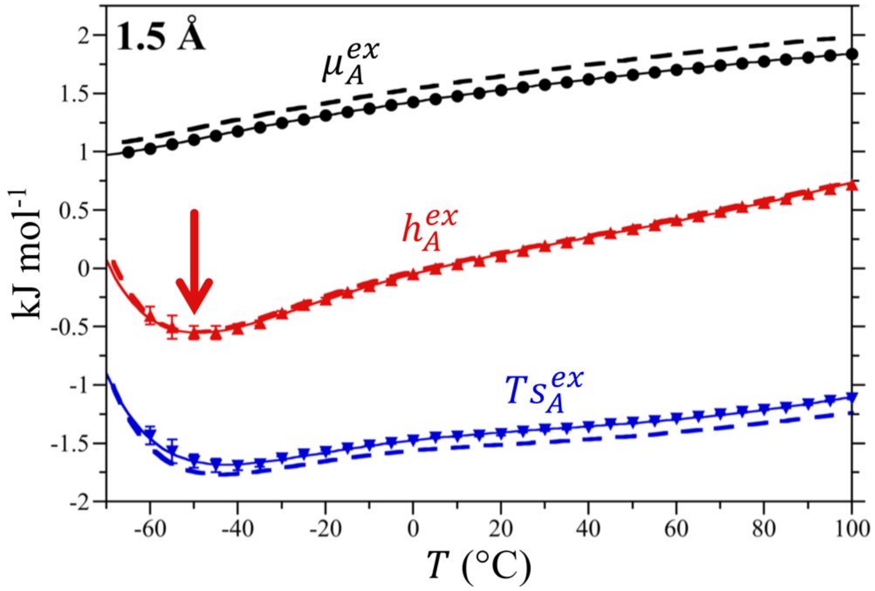

Figure S6. Thermodynamics of dissolution of a 1.5 Å hard sphere solute in water at 1 atm pressure as a function of temperature from -65°C to 100°C. The solid black circles (●), solid red triangles (▲), and solid blue upside-down triangles (▼) correspond the simulation results for the hydration chemical potential, enthalpy, and entropy, respectively. The solid lines (———) correspond to fits of a fifth order polynomial to the simulation chemical potential ($\mu_A^{ex} = \sum_{i=0}^{5} a_i (T_0/T - 1)^i$, where T_0 = 298.15K is a reference temperature) and taking appropriate temperature derivatives to obtain enthalpy and entropy. The red arrow indicates the enthalpy minimum. The simulation error bars correspond to one standard deviation.

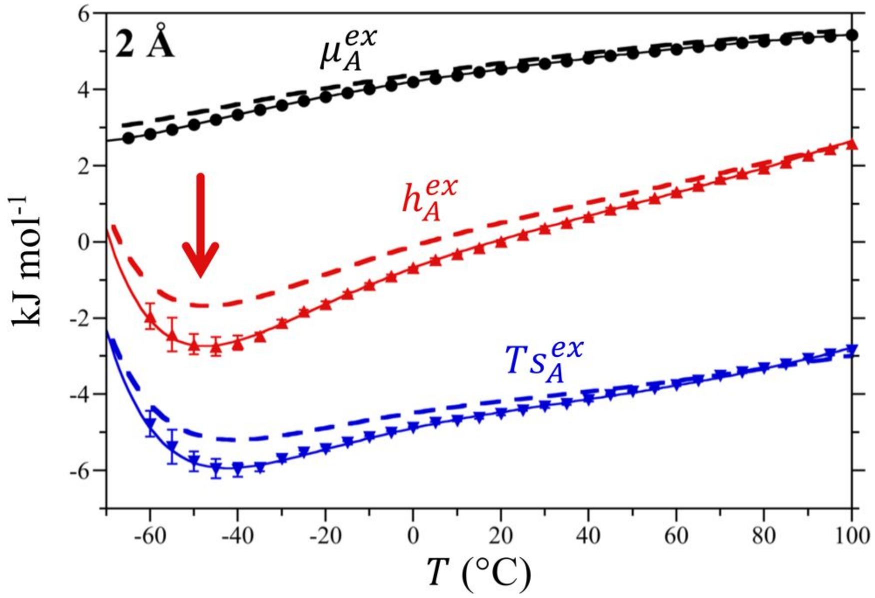

Figure S7. Thermodynamics of dissolution of a 2 Å hard sphere solute in water at 1 atm pressure as a function of temperature from -65°C to 100°C. The solid black circles (●), solid red triangles (▲), and solid blue upside-down triangles (▼) correspond the simulation results for the hydration chemical potential, enthalpy, and entropy, respectively. The solid lines (———) correspond to fits of a fifth order polynomial to the simulation chemical potential ($\mu_A^{ex} = \sum_{i=0}^{5} a_i(T_0/T - 1)^i$, where T_0 = 298.15K is a reference temperature) and taking appropriate temperature derivatives to obtain enthalpy and entropy. The red arrow indicates the enthalpy minimum. The simulation error bars correspond to one standard deviation.

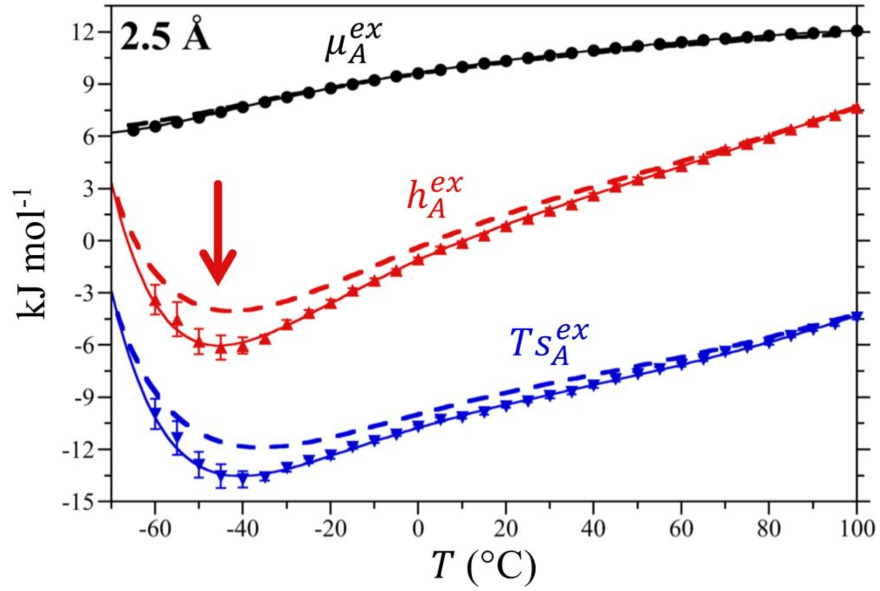

Figure S8. Thermodynamics of dissolution of a 2.5 Å hard sphere solute in water at 1 atm pressure as a function of temperature from -65°C to 100°C. The solid black circles (●), solid red triangles (▲), and solid blue upside-down triangles (▼) correspond the simulation results for the hydration chemical potential, enthalpy, and entropy, respectively. The solid lines (———) correspond to fits of a fifth order polynomial to the simulation chemical potential ($\mu_A^{ex} = \sum_{i=0}^{5} a_i (T_0/T - 1)^i$, where T_0 = 298.15K is a reference temperature) and taking appropriate temperature derivatives to obtain enthalpy and entropy. The red arrow indicates the enthalpy minimum. The simulation error bars correspond to one standard deviation.

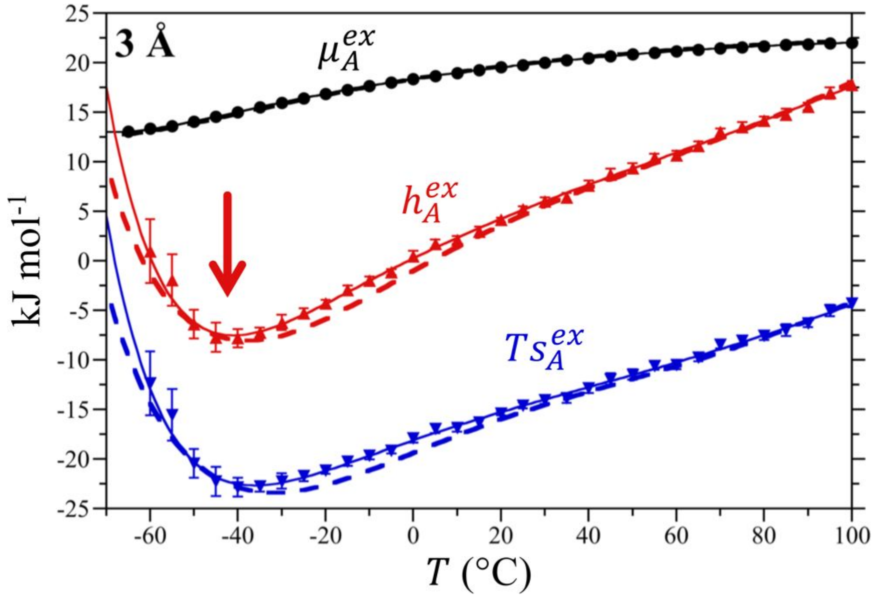

Figure S9. Thermodynamics of dissolution of a 3 Å hard sphere solute in water at 1 atm pressure as a function of temperature from -65°C to 100°C. The solid black circles (●), solid red triangles (▲), and solid blue upside-down triangles (▼) correspond the simulation results for the hydration chemical potential, enthalpy, and entropy, respectively. The solid lines (———) correspond to fits of a fifth order polynomial to the simulation chemical potential ($\mu_A^{ex} = \sum_{i=0}^{5} a_i (T_0/T - 1)^i$, where T_0 = 298.15K is a reference temperature) and taking appropriate temperature derivatives to obtain enthalpy and entropy. The red arrow indicates the enthalpy minimum. The simulation error bars correspond to one standard deviation.

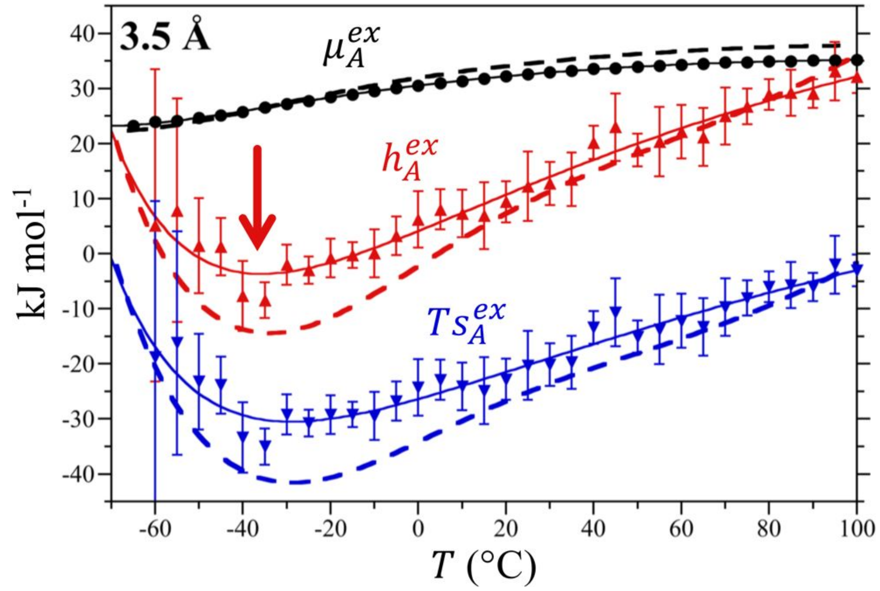

Figure S10. Thermodynamics of dissolution of a 3.5 Å hard sphere solute in water at 1 atm pressure as a function of temperature from -65°C to 100°C. The solid black circles (●), solid red triangles (▲), and solid blue upside-down triangles (▼) correspond the simulation results for the hydration chemical potential, enthalpy, and entropy, respectively. The solid lines (———) correspond to fits of a fifth order polynomial to the simulation chemical potential ($\mu_A^{ex} = \sum_{i=0}^{5} a_i (T_0/T - 1)^i$, where T_0 = 298.15K is a reference temperature) and taking appropriate temperature derivatives to obtain enthalpy and entropy. The red arrow indicates the enthalpy minimum. The simulation error bars correspond to one standard deviation.

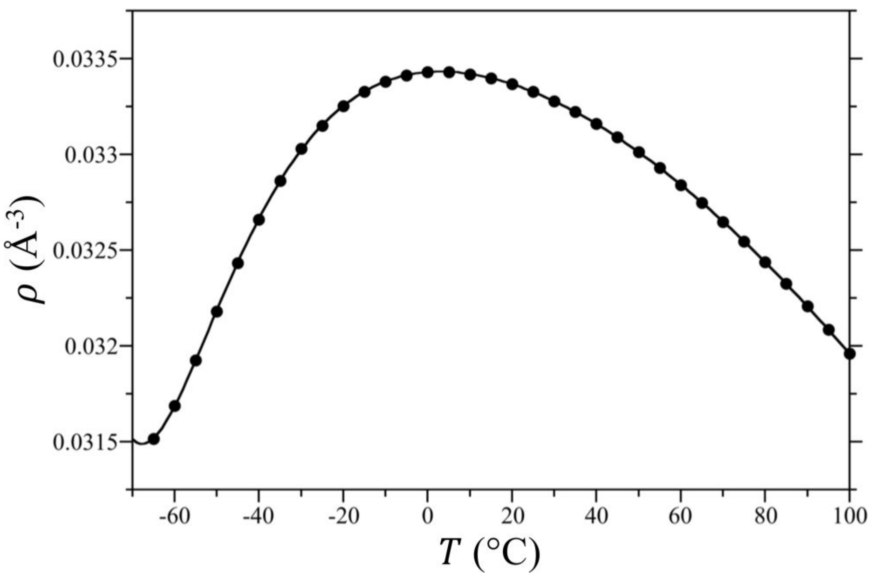

Figure S11. Density of water as a function of temperature from the metastable supercooled liquid regime (-65°C) to the normal boiling point (100°C) at 1 atm pressure. The points correspond to the simulation results. The line corresponds to the fit of eq. (S3) to the simulation data. The simulation error bars are smaller than the symbols.

References

1) M. J. Abraham, T. Murtola, R. Schulz, S. Páll, J. C. Smith, B. Hess, E. Lindahl, "Gromacs: High Performance Molecular Simulations through Multi-Level Parallelism from Laptops to Supercomputers," *SoftwareX* (2015) **1-2**, 19-25.
2) J. L. F. Abascal, C. Vega, "A General Purpose Model for the Condensed Phases of Water: Tip4p/2005," *Journal of Chemical Physics* (2005) **123**, 12.
3) T. Darden, D. York, L. Pedersen, "Particle Mesh Ewald - an N.Log(N) Method for Ewald Sums in Large Systems," *Journal of Chemical Physics* (1993) **98**, 10089-10092.
4) S. Nosé, "A Unified Formulation of the Constant Temperature Molecular-Dynamics Methods," *Journal of Chemical Physics* (1984) **81**, 511-519.
5) W. G. Hoover, "Canonical Dynamics: Equilibrium Phase-Space Distributions," *Physical Review A* (1985) **31**, 1695-1697.
6) M. Parrinello, A. Rahman, "Polymorphic Transitions in Single-Crystals - a New Molecules-Dynamics Method," *Journal of Applied Physics* (1981) **52**, 7182-7190.
7) S. Miyamoto, P. A. Kollman, "Settle - an Analytical Version of the Shake and Rattle Algorithm for Rigid Water Models," *Journal of Computational Chemistry* (1992) **13**, 952-962.
8) B. Widom, "Some Topics in the Theory of Fluids," *Journal of Chemical Physics* (1963) **39**, 2808-2812.
9) K. S. Shing, S. T. Chung, "Computer-Simulation Methods for the Calculation of Solubility in Supercritical Extraction Systems," *Journal of Physical Chemistry* (1987) **91**, 1674-1681.
10) H. S. Ashbaugh, H. Bukannan, "Temperature, Pressure, and Concentration Derivatives of Nonpolar Gas Hydration: Impact on the Heat Capacity, Temperature of Maximum Density,

and Speed of Sound of Aqueous Mixtures," *Journal of Physical Chemistry B* (2020) **124**, 6924-6942.

11) J. D. Weeks, D. Chandler, H. C. Andersen, "Role of Repulsive Forces in Determiningthe Equilibrium Structure of Simpleliquids," *Journal of Chemical Physics* (1971) **54**, 5237-5247.

12) H. Reiss, H. L. Frisch, E. Helfand, J. L. Lebowitz, "Aspects of the Statisticalthermodynamics of Real Fluids," *Journal of Chemical Physics* (1960) **32**, 119-124.
